# Development of a value assessment framework for Health Technology Assessment in rare diseases drugs: insights from a Delphi study in Brazil

**DOI:** 10.1017/S0266462324004835

**Published:** 2025-01-09

**Authors:** Luiza Vasconcelos Biglia, Arturo Felippini, Tatiane Bomfim Ribeiro, Tácio de Mendonça Lima, Patricia Melo Aguiar

**Affiliations:** 1 Department of Pharmacy, Faculty of Pharmaceutical Sciences, University of São Paulo, São Paulo, Brazil; 2 Faculty of Public Health, University of São Paulo, São Paulo, Brazil; 3 Department of Pharmacy and Pharmaceutical Administration, Federal Fluminense University, Niterói, Brazil

**Keywords:** rare diseases, health technology assessment, validation study, value assessment framework, Brazilian Healthcare System

## Abstract

**Objective:**

The aim of this study is to propose and validate a value assessment framework for Health Technology Assessment (HTA) for rare diseases drugs in Brazil.

**Methods:**

A scoping review was performed to identify criteria used by HTA agencies in countries with public healthcare systems when evaluating orphan drugs. Based on the findings, a criteria framework for rare disease drugs was proposed for Brazil. Content validity was conducted over three rounds using Delphi technique and content validity ratio (CVR) approach was employed to evaluate the ratings from the eighteen stakeholders (experts and patients).

**Results:**

Twenty-nine HTA criteria for rare disease drugs were identified to compose the Brazilian framework. After three Delphi rounds, the final value framework comprised fifteen criteria categorized into four domains: disease-related factors, treatment-related factors, social and political factors, and economic factors. Among the most well-rated criteria by the CVR, considering the relevance attribute, were “relevance of outcomes for a rare disease,” “impact on patient’s quality of life,” “price negotiation,” and “adjusted cost-effectiveness threshold.” On the other hand, “budget impact threshold,” “innovative nature of treatment,” and “willingness to accept greater uncertainty in clinical evidence” received negative evaluations and were excluded from the final framework.

**Conclusions:**

A value assessment framework validated by key stakeholders of rare diseases in Brazil could contribute to improve HTA transparency, decision making, and efficiency of the healthcare system, and inspire the development of a local guidance for rare-disease HTA.

## Introduction

In Brazil, the Unified Healthcare System (SUS) was established in 1990 under Law 8,080. The principles of SUS are Universality, Comprehensiveness, and Equity (1), as outlined in Article 196 of the Brazilian Constitution of 1988, which states that “health is a right of all and a duty of the State” (2).

To optimize resource allocation efficiency, the Health Technology Assessment (HTA) committee – known as Conitec – was formed in 2011 by Law 12,401 to support the Ministry of Health in decision making. This legislation outlined the HTA process and set timelines for technology evaluation and incorporation into the public system (3). However, in the rare disease setting, a differentiated HTA process is not yet clearly defined in Brazil or several other countries (4).

Following extensive consultations involving the Ministry of Health, policy makers, researchers, physicians, and patient associations, the National Policy for Comprehensive Care for People with Rare Diseases in SUS was enacted in 2014, defining rare diseases as those with a prevalence below sixty-five per 100,000 people (5). One of the guiding principles of this policy is that the incorporation of drugs for rare diseases, known as orphan drugs, should be determined by the Ministry of Health based on Conitec’s evaluation and recommendation process (5).

As demonstrated by Biglia et al. (4), the establishment of Conitec has improved the landscape of rare diseases in the Brazilian public health system. Over half (52 percent) of the drugs for rare diseases evaluated by Conitec between 2012 and 2019 received a positive recommendation and were subsequently incorporated into the system. Despite this progress, due to the increasing demand for health, maintaining the cost-effectiveness and sustainability of the system is a challenge not only in Brazil but also globally (6). For this reason, it is crucial to debate the most effective strategies for evaluating technologies for rare diseases, beyond cost-effectiveness and budgetary impact (7).

Given the constraints of healthcare budgets, there is a growing imperative to make informed decisions to ensure that the necessary technologies reach the patients. Challenges in HTA for orphan drugs include limited scientific evidence, heterogeneity of rare disease populations, and the high cost of treatments (7;8). Notably, as identified in a previous study (4), Brazil lacks adapted criteria for evaluating the incorporation of rare diseases drugs. Establishing a differentiated value assessment tailored for rare diseases could assist HTA agencies, such as Conitec, in the evaluation of orphan drugs with greater alignment to their unique needs and economic considerations.

On the international scene, agencies in countries such as the United Kingdom, Canada, France, and Australia, which are pioneers in HTA, have developed specialized processes for evaluating and recommending orphan drugs. However, this remains a complex and evolving area of focus (9). Currently, there is no specific framework in place for the HTA of rare diseases in Brazil. The absence of specific guidelines for evaluating health technologies for rare diseases in Brazil is a significant factor that can impact the analyses conducted by Conitec (4). Therefore, the present study aims to propose and validate a value assessment framework for evaluating HTA criteria for rare diseases within the Brazilian public healthcare system.

## Methods

A methodological study was carried out in the Brazilian context from March to June 2023, structured in three steps: (1) identification of potential HTA criteria for rare diseases through a scoping review, (2) proposal of an initial value assessment framework, and (3) validation of the proposed framework using the Delphi technique and statistical analyses.

### Identification of potential HTA criteria for rare diseases

Initially, a scoping review (10) was conducted using databases including PubMed, LILACS, Scopus, and Embase, as well as gray literature sources such as Google Scholar and websites of HTA agencies. The objective was to identify publications addressing the criteria used by HTA agencies in countries with public healthcare systems (both fully public and hybrid) when evaluating reimbursement recommendations for orphan drugs. It is important to note that the definition of criterion adopted in this review refers to any item proposed to standardize the assessment process – whether qualitative, quantitative, or even discussion points. If addressed, these criteria would help minimize information asymmetry and enhance understanding and transparency among stakeholders.

The research question was formulated based on the PCC elements: Population (rare diseases), Concept (specific/differentiated criteria for orphan drug evaluation), and Context (HTA agencies of countries with public healthcare systems). The search resulted in twenty-three articles, published between 2014 and 2023, and covering the following seventeen countries: Argentina, Australia, Brazil, Canada, Finland, France, Germany, Ireland, Italy, the Netherlands, Poland, Russia, South Korea, Spain, Sweden, Switzerland, and the United Kingdom. The mapped criteria were then organized according to the countries’ categorization within one of three models of healthcare systems: National Health System, National Health Insurance, and Social Health Insurance. These countries were chosen following the list of agencies affiliated with the International Network of Agencies for Health Technology Assessment, in order to focus our efforts on centralized national organizations willing to share data.

### Proposal of a value assessment framework for HTA criteria in rare diseases in the Brazilian Public System

The development of HTA criteria within the value assessment framework involved a comprehensive consideration of results from a scoping review (10), which identified key criteria used in public healthcare systems for evaluating rare diseases. The research team, composed of two research professors specializing in HTA and/or validity evidence process, two pharmacist practitioners with experience in HTA and/or rare diseases, and an undergraduate pharmacy student, undertook a thorough analysis to determine which criteria could be adapted for inclusion in the proposed framework for Brazil. To facilitate the understanding and data organization into domains, the methodological structure of the European Network for Health Technology Assessment (EUnetHTA) was used as a theoretical reference (11).

### Content validity of a value assessment framework for HTA criteria in rare diseases in the Brazilian Public System

#### Delphi rounds

The Delphi technique was employed to achieve consensus among a panel of stakeholders using an online questionnaire developed in Google Forms. This method, widely utilized in health research, provides a structured approach to synthesizing expert opinions through iterative rounds of feedback, promoting transparency and inclusivity. This is consistent with HTA practices employed in international value assessment frameworks (12–14). In this study, the Delphi technique was used to evaluate whether the criteria within the framework accurately represented the domains of interest and were suitable for Brazil, through a qualitative and quantitative process. Usually, a panel of five to ten stakeholders is considered sufficient for this assessment (15). Thirty stakeholders with recognized experience and solid knowledge in HTA and/or rare diseases were identified, including former and current members of Conitec and the Ministry of Health, as well as representatives from patient associations, university professors, and researchers in this field, from various regions of Brazil. Stakeholders were invited via email to contribute to the framework, and all those who agreed signed an Informed Consent Form. A questionnaire was administered to collect sociodemographic information from participants (including age, gender, educational degree, area of expertise, length of professional experience, and region of practice), along with their assessment of the initial version of the framework.

A total of three rounds were conducted to gather content validity evidence for the framework. In the first round, the stakeholders panel evaluated three attributes of each criterion proposed in the framework: clarity of language (assessing whether the language used is clear, understandable, and appropriate), theoretical relevance (evaluating the relevance of the items to the underlying theory), and practical pertinence (determining whether the item assesses a concept of interest to the target population). During the round, stakeholders also had the opportunity to provide suggestions related to technical content and grammar (16). Each attribute was rated by the stakeholders using a five-point Likert scale, ranging from 1 (*strongly disagree*) to 5 (*strongly agree*) (17).

The research group reviewed the recommendations and suggestions provided by the stakeholder’s panel, incorporating those considered most pertinent into the framework. Subsequently, a new round of the Delphi method was conducted to evaluate the attributes that had been restructured based on feedback from the first round. The same iterative process occurred between rounds two and three of the assessment. This approach ensured that the framework underwent refinement and content validity through multiple cycles of stakeholders’ evaluation and feedback, enhancing its robustness and relevance for assessing HTA criteria in the context of rare diseases.

#### Data collection and analysis

At each round of the content validity process, the stakeholders’ responses were compiled into Excel® for analysis. Data concerning the stakeholders’ characteristics in each round were analyzed and presented descriptively. To assess potential shifts in diversity throughout the Delphi process, chi-square (χ^2^) tests were applied to categorical variables, and analysis of variance (ANOVA) was used for continuous variables. A significance level of *p* < .05 was considered for all tests. In addition, the agreement among stakeholders regarding the framework was assessed using the content validity ratio (CVR) (18).

The CVR was employed to evaluate the content validity of the HTA criteria by calculating the proportion of stakeholders who considered each attribute as “essential” (rated as 4 or 5 on the Likert scale). A minimum CVR value, corresponding to the probability of type I error, unilateral test with *p* = .05, was determined based on the number of stakeholders involved (19), calculated using the formula:

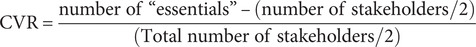



A CVR of 1 indicates unanimous agreement among all stakeholders that the criterion is essential for inclusion. A CVR between 0 and 1 suggests that more than half of the stakeholders considered the criterion essential. Conversely, a CVR between −1 and −3 indicates that more than half of the stakeholders rated the criterion as non-essential. The critical cutoff values for the CVR, used to determine agreement exceeding chance, were .444 for eighteen stakeholders, .667 for twelve stakeholders, and .778 for nine stakeholders (18;19). Items that received a CVR above the specified cutoff value were incorporated into the framework (individually or grouped with another item, depending on the suggestions). In contrast, items that fell below this cutoff were rejected or carried forward to the next round for further consideration.

## Results

### Proposal of a value assessment framework for HTA criteria in rare diseases in the Brazilian Public System

Following a detailed analysis of the findings of the scoping review and aligning the criteria in the respective domains of the EUnetHTA, those best suited to the Brazilian HTA public policies for rare diseases were modified and proposed within a framework consisting initially of five new suggested domains: (1) Disease, (2) Technology, (3) Social Perspective, (4) Jurisprudence, and (5) Economic Evaluation.

Twenty-nine criteria were elaborated, organized, and proposed across these five domains in the framework, designated as the initial version (Supplementary Appendix 1). The domain “Technology” encompassed characteristics, efficacy, and safety of orphan drugs, with nine criteria included (31 percent of the proposed criteria). The “Social Perspective” domain focused on patient, social, and ethical aspects, incorporating seven criteria (24 percent). The “Economic Evaluation” domain included six criteria, representing 20.7 percent of the twenty-nine criteria proposed in the framework. Only three differentiated criteria were included in the “Jurisprudence” domain (10 percent), this domain is related to legal and organizational aspects.

### Content validity of a value assessment framework for HTA criteria in rare diseases in the Brazilian Public System

#### Stakeholders panel

Table 1 presents the characteristics of stakeholders involved in the framework’s content validity process. Out of the thirty stakeholders invited in the first round, eighteen (60 percent) accepted and engaged in the content validity process using the Delphi technique. Response rates were 67 percent (12/18) for the second round and 75 percent (9/12) for the third round. In the first round, most participants identified as cisgender men (10; 55.6 percent), with a mean age of 40.7 years (SD = 13.6). More than half of participants held advanced degrees, such as a master’s, PhD, or post-PhD. The panel represented a diverse distribution across four of the five Brazilian regions. The stakeholder panel predominantly comprised individuals from the HTA area (88.9 percent), bringing an average of 9.7 years of experience (SD = 4.9, range: 3–21 years). Among the panel, 22.2 percent had affiliations with Conitec or Health Technology Assessment Centers (NATS), and 33.3 percent represented patient groups, the pharmaceutical industry, or academia, adding diverse perspectives to the content validity process. No statistically significant differences in participant characteristics across the rounds (*p* > .05 for all variables) were observed.

**Table 1.** Characteristics of the stakeholders who participated in this study

Variable^a^	Round 1 (*n* = 18)		Round 2 (*n* = 12)		Round 3 (*n* = 9)	
	*n*	%	*n*	%	*n*	%
*Age (years), mean (SD)*	40.7	(13.6)	40.2	(15.4)	38.6	(15.4)
*Gender identity*						
Cisgender woman	8	44.4	6	50.0	6	66.7
Cisgender man	10	55.6	6	50.0	3	33.3
*City and state of residence*						
São Paulo – SP	5	27.8	4	33.3	2	22.2
Brasília – DF	5	27.8	3	25.0	3	33.3
Rio de Janeiro – RJ	2	11.1	1	8.3	1	11.1
Florianópolis – SC	2	11.1	1	8.3	0	0.0
Nova Friburgo – RJ	1	5.6	0	0.0	0	0.0
Curitiba – PR	1	5.6	1	8.3	1	11.1
João Pessoa – PB	1	5.6	1	8.3	1	11.1
Porto Alegre – RS	1	5.6	1	8.3	1	11.1
*Area of expertise* ^b^						
HTA	16	88.9	11	91.7	8	88.9
Rare diseases	7	38.9	5	41.7	3	33.3
Patient or group of patients	3	16.7	1	8.3	1	1.1
*Length of service (years), mean (SD)*	9.7	(4.9)	8.0	(3.3)	7.8	(3.3)
*Activity profile*						
I am/was a member of Conitec	4	22.2	3	25.0	2	22.2
I am/was a member of NATS	4	22.2	3	25.0	3	33.3
Researcher in the field	4	22.2	2	16.7	1	11.1
Patient groups	2	11.1	2	16.7	1	11.1
Pharmaceutical industry	2	11.1	2	16.7	2	22.2
University professor	2	11.1	0	0.0	0	0.0
*Level of education*						
Undergraduation	3	16.7	2	16.7	1	11.1
Latu senso specialization	3	16.7	3	25.0	3	33.3
Master	4	22.2	3	25.0	2	22.2
PhD	6	33.3	4	33.3	3	33.3
Post-PhD	2	11.1	0	0.0	0	0.0

Abbreviations: Conitec, National Committee for Health Technology Incorporation in the Unified Health System; NATS, Health Technology Assessment Centers; ANOVA, analysis of variance.

a Chi-square (χ^2^) tests were performed for categorical variables and ANOVA for continuous variables. No significant differences were observed across the rounds (*p* > .05).

b Answers are not mutually excluding; percentages do not complete 100%.

#### Content validity of HTA criteria for rare diseases

**Figure 1.**
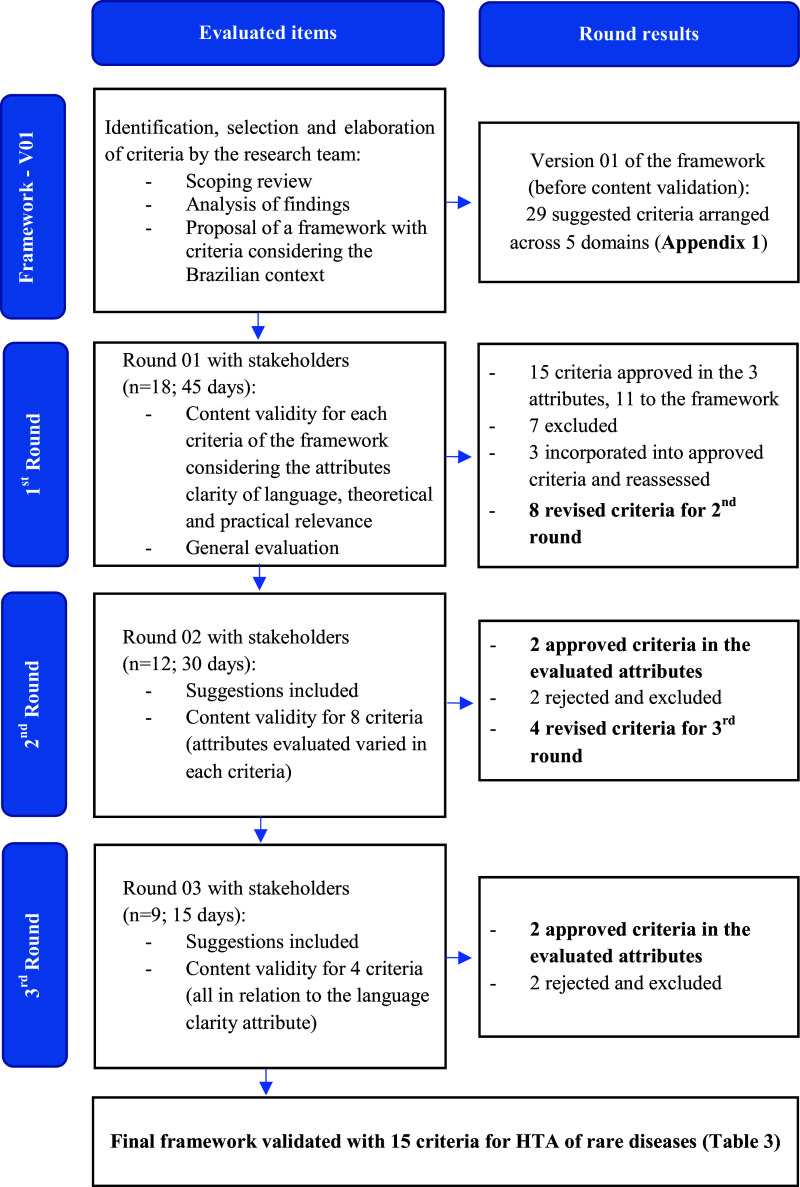
Proposal and content validity process of a value assessment framework for HTA criteria in rare diseases in Brazil. HTA, Health Technology Assessment.

A flowchart illustrating the steps involved in proposing and validating the framework of HTA criteria is presented in Figure 1. In addition, Table 2 provides an overview of the three rounds of content validity conducted for each of the twenty-nine criteria within the framework.

**Table 2.** Content validity assessment of HTA criteria for rare diseases in Brazil

Attributes (CVR)										
	Round 1				Round 2				Round 3	
Criteria proposed and evaluated in the initial version of the framework	Language clarity	Theoretical relevance	Practical pertinence	Decision	Language clarity	Theoretical relevance	Practical pertinence	Decision	Language clarity	Decision
1. Rarity of the disease (can allow the understanding of the nature of the disease considering its prevalence)	0.22	0.67	0.78	Language improvement	0.17	–	–	Language improvement	0.78	Approved
2. Definition of ultra-rare disease	−0.11	0.56	0.67	Excluded	–	–	–	–	–	–
3. Severity of illness (e.g., permanent damage, affects children, affects activities of daily living etc.)	0.22	0.56	0.89	Language improvement	0.67	–	–	Approved	–	–
4. Unmet medical need (lack of available treatment for the condition in the healthcare system)	0.56	0.67	0.89	Approved	–	–	–	–	–	–
5. Facilitated administration	−0.11	0.11	0.44	Excluded	–	–	–	–	–	–
6. Innovative nature of treatment (translates into clinical gains for patients and not just a new class of drugs or mechanism of action)	0.00	0.22	0.33	Language improvement	0.33	0.67	0.67	Language improvement	0.56	Excluded
7. Need for training of professionals and caregivers	0.78	0.67	0.67	Approved but reevaluated with 8	–	–	–	–	–	–
8. Impact of technology on the use of health system resources (e.g., need for training of professionals and caregivers, changes in health system infrastructure, etc.)	0.33	0.33	0.33	Language improvement	0.33	1.00	0.83	Language improvement	1.00	Approved
9. Relevance of outcomes for a rare disease (e.g., consensus among HTA technicians, physicians, patients, literature, including willingness to accept surrogate endpoints, etc.)	0.44	0.78	0.78	Approved	–	–	–	–	–	–
10. Impact on patient’s quality of life (e.g., well-being from perceived symptom improvement)	0.78	0.78	0.67	Approved	–	–	–	–	–	–
11. Type of treatment benefit (curative, palliative, or preventive)	0.78	0.44	0.56	Approved	–	–	–	–	–	–
12. Willingness to accept greater uncertainty in clinical evidence (e.g., from non-randomized clinical trials)	0.33	0.44	0.44	Language improvement	0.17	–	–	Language improvement	0.56	Excluded
13. In case of uncertainty of the evidence, consider the possibility of making the drug available for a certain period with the commitment that the manufacturer will collect efficacy data from patients using the medication for HTA reassessment	0.89	0.56	0.22	Excluded	–	–	–	–	–	–
14. Patient participation in the decision-making process	0.44	0.78	0.56	Approved but reevaluated with 23	–	–	–	–	–	–
15. Participation of society in the decision-making process	0.33	0.56	0.22	Excluded	–	–	–	–	–	–
16. Participation of disease specialists in the decision-making process	0.44	1.00	0.78	Approved but reevaluated with 23	–	–	–	–	–	–
17. Social aspects for patients (e.g., return to work or school, psychosocial impact, possibility of performing daily activities when treated, etc.)	0.89	0.89	0.67	Approved	–	–	–	–	–	–
18. Social aspects for caregivers and family members (e.g., possibility of work, psychosocial impact, etc.)	0.89	0.78	0.67	Approved	–	–	–	–	–	–
19. The treatment allows the patient to contribute to society again and resume daily activities	0.89	0.56	0.44	Approved but similar to 10	–	–	–	–	–	–
20. Impact of treatment on the distribution of health care to the population (ethical dilemmas regarding the magnitude of the effect and distributive justice)	−0.22	−0.11	−0.33	Language improvement	0.00	0.50	0.33	Excluded	–	–
21. Public policies for prioritizing the rare condition/disease (e.g., whether or not the disease is part of a public prioritization policy)	0.44	0.44	0.67	Approved	–	–	–	–	–	–
22. Clear reduction in the use of health system resources	0.44	0.44	0.33	Excluded	–	–	–	–	–	–
23. Committee with different actors to advise the HTA technician in the process of understanding the disease (e.g., clinical specialists in the care of the disease, geneticists, reference centers, etc.)	0.78	0.33	0.44	Language improvement	–	0.67	–	Approved	–	–
24. Adjusted budget impact threshold (e.g., depending on disease rarity, effect magnitude, etc. – possibly within a predefined range)	1.00	0.67	0.33	Language improvement	–	–	0.50	Excluded	–	–
25. Adjusted cost-effectiveness threshold (e.g., depending on disease rarity, magnitude of effect, etc. – possibly within a predefined range)	0.56	0.56	0.56	Approved	–	–	–	–	–	–
26. Risk sharing between manufacturer and payer (e.g., manufacturer follows up with patients and commits to data publication)	0.67	0.56	0.56	Approved	–	–	–	–	–	–
27. Price confidentiality	0.00	−0.22	−0.22	Excluded	–	–	–	–	–	–
28. Possibility of selecting the population with the greatest benefit (e.g., from pre-specified subgroups and outcome drivers)	0.56	0.56	0.44	Approved	–	–	–	–	–	–
29. Price Negotiation	0.78	0.89	0.89	Approved	–	–	–	–	–	–

*Note:* The cutoff for eighteen responders is ≥.444, for twelve responders is ≥.667 and for nine responders is ≥0.778 (19).

Abbreviations: CVR, content validity ratio; HTA, Health Technology Assessment.

In the first round of the content validity process, the CVR for the evaluated attributes (language clarity, theoretical relevance, and practical pertinence) of each proposed criterion ranged from −.333 to 1, with a critical CVR value of .444 for 18 stakeholders. Fifteen of the twenty-nine proposed criteria (51.7 percent) were approved in all evaluated attributes, while four (13.7 percent) failed in all attributes. However, only eleven criteria were directly incorporated into the framework. Three required modifications and reevaluation and one was excluded for being considered equivalent to another approved criterion, according to the stakeholders.

For the second round of the content validity process, seven criteria were excluded, and eight criteria were modified according to the stakeholders’ interpretation. Three of the criteria that were reevaluated in this round had already reached an agreement in the previous round. However, changes were made to improve language clarity and to group excluded criteria, due to the similarities in theme. For this reason, these criteria were reassessed. For those criteria that were not approved, it is interesting to mention that “Price Confidentiality” was excluded due to high agreement among stakeholders that it would not work in the Brazilian model (CVR between 0 and −0.22).

Twelve stakeholders participated in the second round, meeting the critical CVR value of .667. Two out of the eight criteria evaluated were approved at this stage. After interpreting the stakeholders’ assessments, another two criteria were excluded, as was the case with the “Budget impact threshold.” Stakeholders expressed concern about this criterion becoming a limitation in decision making. Four criteria passed to the third round. The only attribute to be evaluated in the third round was language clarity.

Further refinements were made to enhance criteria comprehension. Of the four criteria, two reached the critical value of CVR, which is .778 for nine respondents. Therefore, “Severity of the disease” and “Impact of technology on the use of health care resources” became part of the framework. The other two criteria “Innovative nature of the treatment” and “Willingness to accept greater uncertainties in clinical evidence” despite having reached agreement among stakeholders regarding theoretical relevance and practical relevance, there was no consensus on how these criteria could be described in a framework and, for this reason, were excluded.

After three rounds of content validity through a Delphi panel involving eighteen, twelve, and finally nine stakeholders, the initial framework of twenty-nine proposed criteria was refined to fifteen differentiated criteria, organized into four domains: Disease-related factors, Treatment-related factors, Political and social factors, and Economic factors (Table 3), facilitating the evaluation of health technologies for rare diseases.

**Table 3.** Final value assessment framework for HTA criteria for rare diseases in Brazil

Disease-related factors	Treatment-related factors	Political and social factors	Economic factors
Rarity of the disease (can allow the understanding of the nature of the disease considering its prevalence)^a^	Impact of technology on the use of health system resources (e.g., need for training of professionals and caregivers, changes in health system infrastructure, etc.)	Social aspects for patients (e.g., return to work or school, psychosocial impact, possibility to perform daily activities when treated, etc.)	Adjusted cost-effectiveness threshold (e.g. depending on disease rarity, magnitude of effect, etc. – possibly within a predefined range)
Severity of the disease (e.g., permanent damage, affects children, affects activities of daily living, etc.)	Relevance of outcomes for a rare disease (e.g., consensus among HTA technicians, physicians, patients, literature, including willingness to accept surrogate endpoints, etc.)	Social aspects for caregivers and family members (e.g., possibility of work, psychosocial impact, etc.)	Risk sharing between manufacturer and payer (e.g., manufacturer follows up with patients and commits to data publication)
Unmet medical need (lack of available treatment for the condition in the healthcare system)	Impact on patient’s quality of life (e.g., well-being from perceived symptom improvement)	Public policies for prioritizing the rare condition/disease (e.g., whether or not the disease is part of a public prioritization policy)	Possibility of selecting the population with the greatest benefit (e.g., from pre-specified subgroups and outcome drivers)
	Type of treatment benefit (e.g., curative, palliative, or preventive)	Committee with different actors to advise the HTA technician in the process of understanding the disease (e.g., clinical specialists in the care of the disease, geneticists, reference centers, etc.)	Price Negotiation

Abbreviation: HTA, Health Technology Assessment.

a The disease rarity criteria aims to make the framework more flexible for a variety of interpretations that may be considered with the concept of “rare disease.” It is not the intention of this work to determine how this could be done, but rather that it is a point that must be considered in the context of a differentiated HTA assessment.

## Discussion

Implementing a value assessment framework for rare diseases presents significant challenges, mainly due to the diverse nature of healthcare systems across countries and the intricate complexities inherent to these diseases. According to Novaes et al. (20), there is a need for a coherent value framework that encompasses all attributes relevant to health technologies, reflecting both social preferences and legal commitment assumed by institutions. Considering this context, a set of specific criteria tailored for Brazil was proposed and validated in three rounds of the Delphi panel involving eighteen Brazilian stakeholders.

When comparing the proposed framework for Brazil with international criteria identified in our scoping review (10), one of the main similarities is the emphasis on addressing unmet medical needs, rarity, and severity of diseases, common in countries such as Australia, Canada, England, and others in Europe. Adjusted cost-effectiveness thresholds and collaborative stakeholder involvement are also practices seen in nations, such as Australia, England, France, and Wales. On the other hand, some criteria adopted in other countries, such as accepting higher levels of evidence uncertainty, adjusted budget impact thresholds, and prioritizing treatment innovation were not included in our framework.

The proposition of a framework with specific or adapted criteria for evaluating health technologies for rare diseases can serve as guiding material for future discussions. This could include the development of a manual for evaluating rare disease drugs, similar to those already available on Conitec’s website (e.g., the Guideline for the Economic Evaluation and Budget Impact Analysis) (21). Such tailored guidance holds the potential to enhance transparency and reduce bias in the assessment process, addressing the pressures faced by HTA agencies (20). Notably, in Europe, new programs specific to rare diseases drugs have been implemented, like the *Highly Specialised Technology* (HST) in the United Kingdom, which provides a manual for the evaluation of reimbursement recommendations for rare diseases (22, 23), emphasizing the importance of transparent processes. In the future, innovative strategies may emerge to refine the utilization of the proposed criteria in our framework and expedite the decision-making process.

The initial framework proposed for content validity by experts encompassed twenty-nine criteria, which were assessed based on three attributes: language clarity, theoretical relevance, and practical pertinence. Approval rates were promising, with more than half of the criteria gaining acceptance, leading to the inclusion of eleven criteria in the framework following the initial round of evaluation. This outcome suggests a positive inclination toward the necessity of tailored criteria for rare diseases. Following the stakeholders’ evaluation, four approved criteria underwent modifications and were subsequently reevaluated in the second round. Notably, one criterion, “Price confidentiality,” was excluded in the first round, due to a negative CVR, which ranged from −.22 to 0. The stakeholders cited Brazilian legislation mandating the publication of public procurement prices (24), contrasting with practices in other countries like the United Kingdom, where price negotiations are kept confidential as part of National Institute for Health and Care Excellence (NICE)’s cost control measure (25). Given the importance of this criterion for both payers and society, it could be important to ponder about a strategic system of value-based tiered pricing in order to improve access, enhance efficiency, and empower the country to negotiate with product manufacturers (26).

Despite the exclusion of confidentiality of pricing from the framework for rare diseases, the inclusion of “price negotiation” within the HTA process was immediately approved by the stakeholders. In Brazil, there is no specific discussion regarding pricing with the manufacturer in the HTA process, other than the Public Consultation. However, this does not constitute a comprehensive discussion addressing the needs of both the payer and the manufacturer. Including this possibility in the HTA process could be beneficial in the context of rare diseases, similar to Canada, England, France, Germany, and Ireland (10).

The second round resulted in the approval and inclusion of two additional criteria into the framework. Interestingly, the “Adjusted budget impact threshold” did not receive approval in the practical pertinence attribute and was excluded, despite being approved in the previous round for other attributes. Some stakeholders who negatively rated this attribute expressed concerns about the feasibility of a budget impact range that could constrain HTA assessment. On the other hand, stakeholders who viewed the budget impact threshold more positively emphasized the necessity of delineating financial impacts to guide decision making. In 2017, NICE and the NHS initiated a Public Consultation (27) regarding revisions to the HST program, focusing on evaluation and funding matters. Among the proposed revisions was the introduction of a £20 million “Budget impact threshold,” prompting subsequent studies to assess the impact of this measure (28, 29). Countries, such as England, France, Germany, and the Netherlands, use adjusted budget impact thresholds (10), highlighting a shared approach to balancing cost-effectiveness with the financial impact of rare disease treatments.

In the third and final round of content validity, all criteria were evaluated solely for language clarity. Throughout all three rounds, the most significant challenge was succinctly and clearly translating the complexity of each proposed criterion. Unfortunately, the two criteria “Innovative nature of the treatment” and “Willingness to accept greater uncertainty in clinical evidence” were not approved and were consequently excluded from the final framework. Despite the approval of the attributes of theoretical relevance and practical pertinence, consensus could not be reached regarding language clarity.

The “innovative nature of treatment” for rare diseases is noted particularly in England, France, Italy, Wales, and Sweden (10). One possible explanation for this lack of consensus in our study may be the adoption of NICE’s concept for the innovation criterion. As Nicod et al. (30) suggest, differing national interpretations in accounting for health innovation may have contributed to discomfort among the stakeholder panel. Considering the often scarce evidence for rare diseases, countries, such as Australia, England, France, Germany, Sweden, and Scotland, accept “greater uncertainty in clinical evidence” and emphasize the importance of real-world data in the context of rare diseases (10). The intention behind this criterion in the proposed framework was to introduce the concept of flexibility rather than stipulate the types of clinical studies to be accepted; however, this approach resulted in diverse interpretations and expectations among the stakeholders.

After the three rounds of content validity, a framework comprising fifteen criteria was approved, organized into the following four domains: “Disease-related factors,” “Treatment-related factors,” “Political and social factors,” and “Economic factors.” Despite advancements, uncertainties still abound in the field of HTA, especially those related to rare diseases. Debates on this topic are intensifying among leading researchers from key agencies and certain criteria have gained prominence, such as understanding unmet medical needs, disease nature, as well as different thresholds of willingness to pay and budget impact (10). However, a core set applicable model for HTA agencies has yet to emerge, precisely due to the intrinsic particularities of each country and its healthcare system.

It is important to highlight that in 2021 the General Controller of the Union published an audit of the HTA process in Brazil and found that there is currently no assessment of the SUS’s capacity to financially support the calculated budgetary impact; therefore, there is a recommendation to implement a mechanism aimed at evaluating this capacity for new incorporations (31). Considering that the “Risk Sharing” and “Price Negotiation” criteria were approved and included in the framework, the reflection on the real purchasing capacity of the SUS may be relevant so that access is achieved after incorporation.

It is also worth highlighting that in 2022 Conitec approved a proposal to use cost-effectiveness thresholds in health decisions, with 1 GDP/capita for prevalent diseases and up to 3 GDP/capita for rare diseases (32). In line with the criteria approved in our framework “Adjusted cost-effectiveness threshold”; interesting to note that this Conitec discussion took place simultaneously with this research.

In the last 12 years, Conitec’s efforts have significantly reshaped the landscape of HTA in Brazil. Notably, there has been a concerted push towards enhancing the process, marked by increased transparency, greater social participation, revisions to the decision-making committee’s composition, and the establishment of new committees, among other initiatives. Despite these advancements, several significant technical challenges persist. For example, evaluating cost per quality-adjusted life-year (QALY) poses limitations, as it may not fully capture certain benefits, in addition to biases inherent to less treatable diseases and determining appropriate thresholds (25). A novel approach could involve testing the impact of spillover benefits and related savings that treatments for orphan diseases can have, extending beyond the healthcare sector and profoundly affecting the lives of families dealing with rare diseases. This study emphasizes the urgent need to address these challenges, recognizing them as key points in the ongoing HTA discourse.

While conventional HTA methods are valuable for enhancing healthcare effectiveness and efficiency, they often fail to address the social demands of rare diseases (20). To strive toward universality, comprehensiveness, and equity, aligning with doctrinal principles of the Brazilian public health system (1), continual adjustments and improvements in the HTA process are essential. Ensuring transparency, clarity in criteria and parameters adopted, and management of uncertainties are fundamental conditions for health agencies and institutions to gain societal trust and legitimacy (33).

This study has contributed to the initial discussion on establishing a framework for evaluating health technologies for rare diseases in Brazil, but some limitations must be recognized. Firstly, although a scoping review was conducted to ensure comprehensive criteria development, there remains a possibility that some relevant aspects were overlooked or inadequately captured. In addition, we focused on criteria used in public systems (both fully public and hybrid systems – considering only public aspects), and the exclusion of criteria relevant to private healthcare systems may limit its applicability, especially considering the growing role of private insurance in Brazil. Despite efforts to incorporate diverse perspectives through the Delphi panel, including patients, the pharmaceutical industry, and members of Conitec, their opinions may not be generalized, and the involvement of additional stakeholders might have yielded a different final framework. The reliance on subjective judgments in the evaluation process could also introduce bias. Finally, the framework was tailored to Brazil’s public healthcare system and may require adaptations for use in countries with different regulatory environments or healthcare models.

Future research should focus on the implementation and impact of the proposed HTA criteria framework for rare diseases, as this study was dedicated to its development and validation. It would be interesting to assess these issues from qualitative research – such as interviews or focus groups with local stakeholders: healthcare professionals, patients, and policy makers – that could provide the identification of specific challenges and opportunities for implementing this framework, as well as explore the interest of the Conitec members in developing a tailored model for Brazil. In addition, conducting a pilot study or simulations could be valuable in assessing the potential impact of adopting the framework in the Brazilian context, using evaluation methods, such as cost-effectiveness modeling and budget impact analysis. There is also a need to improve the diversity of stakeholders in future studies by including additional patient groups and industry representatives to ensure that a broader range of perspectives is integrated into the decision-making process.

## Conclusion

This study serves as an initial stage in the discussion toward the establishment of criteria pertinent to HTA for rare diseases in Brazil. Through a comprehensive process involving three rounds of the Delphi panel with the participation of eighteen Brazilian stakeholders, a validated value assessment framework comprising fifteen criteria for rare diseases was developed. While it is recognized that some of these criteria are informally integrated into Conitec’s evaluation process, they are not officially listed in any local HTA manual. This lack of formal recognition may compromise transparency and introduce bias into the process of evaluation of reimbursement recommendations for rare diseases. The findings of this study hold promise for influencing health policy and guiding future research, promoting a more inclusive approach to assessing the accessibility of health technologies for rare diseases.

## Supporting information

Biglia et al. supplementary material Biglia et al. supplementary material

## Supplementary material

The supplementary material for this article can be found at http://doi.org/10.1017/S0266462324004835.
