# Supplementary material for: Development of a value assessment framework for Health Technology Assessment in rare diseases drugs: insights from a Delphi study in Brazil
Source: Int J Technol Assess Health Care. 2025 Jan 9;41(1):e6. doi: 10.1017/S0266462324004835 (PMC11811954; doi:10.1017/S0266462324004835)
Supplement: Biglia et al. supplementary material [file S0266462324004835sup001.docx]

**Appendix 1.** Initial value assessment framework for HTA criteria for rare diseases in Brazil.

| **DISEASE** |  | **TECHNOLOGY** | |  | **SOCIAL PERSPECTIVE** |  | **JURISPRUDENCE** |  | **ECONOMIC EVALUATION** |
| --- | --- | --- | --- | --- | --- | --- | --- | --- | --- |
| **1. Health Problem and Current Technology Use (CUR)** |  | **2. Description and technical characteristics of the technology (TEC)** | **3.Efficacy (EFF) and Safety (SAF)** |  | **4. Patient, Social and Ethical Aspects (SOC) (ETH)** |  | **5. Legal and Organizational Aspects (LEG) (ORG)** |  | **6. Costs and Economic Evaluation (ECO)** |
| Definition of rare disease |  | Facilitated Administration | Definition of relevant outcomes (e.g., understanding of the disease with HTA technicians, prescribers, patients, and literature) |  | Patient Participation |  | Public policies for prioritizing the condition/disease |  | Budget impact threshold (e.g., spending cap over a period, such as 3 years or 5 years) |
| Definition of ultra-rare disease |  | Innovative mechanism of action that impacts the drug safety or efficacy | Impact on quality of life (e.g., reduction of chronic pain, ability to perform activities of daily living when treated) |  | Society Participation |  | Clear reduction in the use of health system resources |  | Adjusted cost-effectiveness threshold |
| Definition of disease severity (e.g., permanent damage, age, risk of death, dependence) |  | Need for training of professionals and caregivers | Type of treatment benefit (curative, palliative, or preventive) |  | Expert Participation |  | Technology evaluation committee with different representations/participants (e.g.: prescribers, geneticists, caregivers, patients, and manufacturers) |  | Risk-sharing (e.g., manufacturer follows up with patients and commits to data publication) |
| Unmet medical need (no treatment available for the condition in the healthcare system) |  | Changes in logistical aspects of the health system | Possibility of flexibility in the uncertainty of scientific evidence |  | Social aspects for patients (e.g., quality of life, possibility of working, leisure, mobility, self-sufficiency, etc.) |  |  |  | Prices Confidentiality |
|  |  |  | In case of uncertainty of the evidence, possibility of making the drug available for a certain period with the commitment that the manufacturer will collect efficacy data from patients using drugs for new HTA |  | Social aspects for caregivers (e.g. quality of life, possibility of working, leisure, psychosocial impact, etc.) |  |  |  | Possibility to select the population with the greatest benefit |
|  |  |  |  |  | The treatment allows the patient to contribute to society again and perform daily activities |  |  |  | Possibility of price reduction |
|  |  |  |  |  | Unmet medical need and magnitude of the effect of technology are so relevant that ethical aspects can be discussed |  |  |  |  |
